# Altered phenotype and gene expression of regulatory T cells (Tregs) in children with Autism, and the relationship with comorbid gastrointestinal symptoms

**DOI:** 10.1186/s12974-026-03701-w

**Published:** 2026-02-17

**Authors:** Rachel J Moreno, Destanie Rose, Paul Ashwood

**Affiliations:** 1 Department of Medical Microbiology and Immunology, UC Davis, UC Davis, CA USA; 2 M.I.N.D. Institute, 50th Street Sacramento, UC Davis, CA 95817 USA

**Keywords:** Autism, Immune, Regulatory t cells, Tregs, Inflammation, Behavior, Gut, GI, Metabolism, Gene expression

## Abstract

Autism Spectrum Disorder (ASD) is a neurodevelopmental condition characterized by social deficits and stereotypic behaviors. In ASD, increased numbers of inflammatory cells and their mediators have been found in peripheral blood, brain, and gastrointestinal (GI) tissues. Regulatory T cells (Tregs) play a crucial role in suppressing inflammatory processes that, if disrupted, can result in inflammation and development of a variety of immunological conditions. In this study, we sought to characterize Tregs populations using flow cytometric and gene transcriptomic approaches in children with ASD (*n* = 36) and typically developing (TD) children (*n* = 18) enrolled in the Childhood Autism Risk from Genetics and Environment (CHARGE)-back study. We also examined differences in the frequencies of activated Tregs in ASD groups when stratified by stable co-occurring GI status. The frequency of gut homing α4β7^+^Tregs positive for inhibitory receptors GITR, LAP, and/or GARP were altered based on the presence of GI symptoms. Analysis of mRNA isolated from CD4^+^CD25^+^Tregs, showed 213 differentially expressed genes (DEGs) between the ASD and TD children. Upregulated DEGs were enriched in Gene Ontology (GO) Biological Processes involved in epigenetic regulation including ‘chromatin organization’, whereas Kyoto Encyclopedia of Genes and Genomes (KEGG) pathways were involved in metabolism, including ‘lipid and atherosclerosis’ and ‘pantothenate and CoA biosynthesis’. Upregulation of immune signaling genes (MAPK3, JAK2, and CASP3) was also noted in ASD Tregs. Downregulated DEGs consisted of genes enriched in immune terms including the GO term ‘leukocyte differentiation’ and KEGG pathways ‘Parkinson disease’ and ‘protein processing in endoplasmic reticulum’. Behavioral correlation analysis pointed to a potential relationship between inappropriate speech scores and lower frequencies of Tregs. Overall, these data support the hypothesis of altered Tregs cell biology in ASD that ultimately may drive a lack of control of inflammatory immune responses in ASD shifting from a balanced state to a dysregulated/inflammatory state.

**Supplementary Information:**

The online version contains supplementary material available at 10.1186/s12974-026-03701-w.

## Introduction

Autism spectrum disorder (ASD) is a behavioral disorder characterized by restrictive and repetitive behaviors, as well as communication and social deficits [1]. The prevalence of ASD continues to rise from what used to be a rare diagnosis 20 years ago to nearly 3% of the population, with 1 in 31 children in the US now diagnosed [1]. This increase may be the result of diagnostic criteria expanding over time and including previously understudied groups. However, improvements in diagnostic assessments are not likely the sole reason for increased diagnostic rates. Causes of ASD have been attributed to genetic pre-dispositions, increased environmental exposures to noxious agents, and the transmission of an altered epigenome throughout generations. Many of the environmental and genetic factors considered to contribute to ASD etiology are also involved in regulating the immune system [2]. For example, genes that are related to immune activation (e.g. mTOR and PTEN) are commonly implicated in syndromic ASD cases [3, 4]. Moreover, ASD linked environmental exposures early in life, such as stress, maternal asthma, exposure to gestational infections, or maternal dysbiosis have also been found to impact the immune system [5–7]. Aberrant immune activity or inflammation is frequently observed in individuals with ASD, with strong evidence suggesting that the severity/degree of immune activation is associated with increased impairments in behavior [8, 9]. Heightened immune activity may leave individuals with ASD more at risk of developing immune mediated comorbidities such as autoimmunity, allergies and gastrointestinal (GI) issues. This is exemplified by the fact that ASD individuals are 6 to 8 times more likely to develop GI issues, or an increased prevalence of autoimmune conditions such as psoriasis [10–13].

Immune dysregulation, particularly T cell dysfunction, is central to many of the co-morbidities that children with ASD are at risk of developing [14]. Increased lymphocyte activation, proliferation, effector programs and proinflammatory cytokines have been observed in children with ASD compared to typically developing (TD) children [14, 15]. Moreover, previous studies suggested that T cell phenotypes in ASD differ depending on the presence of GI issues. For example, children with ASD with GI issues had elevated T helper (T_H_)17 and T_H_17.1 populations, whereas children with ASD without GI symptoms had increased frequencies of T_H_2 populations [16, 17]. Children with ASD with GI issues also had different cytokine responses after immune stimulation, and the degree of the response was related to more impaired behavioral outcomes compared to children with ASD without GI issues [16]. Immune dysregulation, however, is not restricted to T cells, as many cells of the innate immune system are more pro-inflammatory in ASD in general, and further altered in those with GI issues [18–20]. Collectively, these data implies that immune activation occurs in children with ASD and may drive different outcomes in ASD individuals such as co-morbid features. 

Regulatory T cells (Tregs) are essential for maintaining immune tolerance against self-proteins, controlling the immune response and limiting collateral damage following infections [21]. Tregs regulate immune activity via soluble factors and through cell-contact dependent inhibitory mechanisms. Tregs secrete anti-inflammatory cytokines such as transforming growth factor (TGF)β1, interleukin (IL)-10 and IL-35 that have broad effects on the local environment, including downregulation of co-stimulatory receptors and inflammatory cytokine production in innate cells and inhibiting T effector cell programs and T effector cell activation. Reduced plasma levels of anti-inflammatory TGFβ1 and IL-35 are seen in children with ASD and have previously been associated with worse scores on the aberrant behavior checklist (ABC) assessment [22, 23]. In the gut, reduced frequencies of IL-10^+^CD3^+^ T cells were observed in ASD and associated with increased mucosal inflammation [24]. Furthermore, there are a variety of cell-contact dependent inhibitory receptors on Tregs that engage to suppress the immune responses, such as cytotoxic T-lymphocyte associated protein 4 (CTLA-4), which inhibits T effector cell stimulation by competing for binding of CD80/CD86 on antigen presenting cells. Lower frequencies of putative Tregs have been reported in the peripheral blood of ASD children [17, 24–26]. However, the exact Tregs phenotypes reported varied between studies. Forkhead box protein P3 (Foxp3) is a transcription factor whose expression is enriched in Tregs. In ASD, Foxp3^+^ expression is altered, and polymorphisms in the Foxp3 promoter and miRNAs that regulate its expression have been described [27–31]. Cell surface marker IL-2 receptor a chain (CD25^+^) is also associated with Tregs populations. In children with ASD, CD25 fails to be upregulated upon stimulation and frequencies of CD4^+^CD25^+^ cells are decreased [32]. Collectively, these data suggest there may be a dysregulation in Tregs biology in ASD that could contribute to increased inflammation and more behavioral impairments.

Despite their apparent role in ASD pathology, Tregs have not been extensively studied in ASD. Characterization of this regulatory population can provide important insights into the inflammatory processes that occur in ASD. In this study, we examined Tregs isolated from the peripheral blood of children with ASD who were previously enrolled in the Childhood Autism Risk from Genetics and Environment (CHARGE) study and were re-recruited at early/middle childhood (CHARGE-back study) to assess Tregs phenotype markers using flow cytometric and gene expression by high throughput sequencing methods. We also stratify our analysis in the ASD groups to those with and without stable GI issues, observed at both CHARGE and CHARGE-back timepoints, to identify differences in Tregs in the presence of gastrointestinal co-morbidities.

## Methods

### Subject information

Participants for this study were recruited through UC Davis MIND Institute and had all been previously enrolled in the Childhood Autism Risk from Genetics and Environment (CHARGE) study [12]. Participants were randomly recruited from the CHARGE database based on inclusion/exclusion criteria (see below) and typically developing (TD) children were frequency matched to ASD cases based on age, sex and birth location. Previous data taken during enrollment in CHARGE were used to initially screen for inclusion criteria, families were contacted only if they had given consent to be contacted about further studies. Children enrolled in the CHARGE study were grouped in either ASD (*n* = 36, 8 female) or typically developing (TD) (*n* = 18, 2 female) groups (Table 1). Children with ASD received their diagnosis using the Autism Diagnostic Observation Schedule (ADOS) and the Autism Diagnostic Interview-Revised (ADI-R) under the guidance of trained UC Davis MIND institute clinicians. TD children were screened using the Social Communication Questionnaire (SCQ), with scores below 15 confirming their TD status. In all children, the ABC assessment was used to evaluate aberrant behaviors, including irritability, lethargy, stereotypic behavior, hyperactivity, and inappropriate speech. Furthermore, the Mullen Scales of Early Learning (MSEL) and the Vineland Adaptive Behavior Scales (VABS) assessments were used to gain insights into secondary behaviors and traits. At the time of recruitment into the CHARGE study, the parents or legal guardians of the study participants were asked to complete the CHARGE GI history (GIH) survey, based upon Rome III Diagnostic Questionnaire for the Pediatric Functional GI Disorders, that rates the frequency of GI symptoms on a Likert scale [(0) = never, (1) = rarely, (2) = sometimes, (3) = frequently and (4) = always)] [10]. To help determine if children with ASD had stable GI symptoms (ASD^GI^, *n* = 12) or did not have any GI symptoms at either timepoint (ASD^NoGI^, *n* = 24), parents and legal guardians completed the CHARGE GI history (GIH) assessment again on enrollment in CHARGE-back. The GIH is a validated tool used in previous publications [13, 16]. The GIH evaluates the current frequency of GI symptoms within the past 3 months, such as abdominal pain, gaseousness/bloating, constipation, diarrhea, difficulty swallowing, blood in stool/vomit, food sensitivity, and vomiting. Parents/guardians were also asked if the participants experienced any allergies to foods, if their diet was restricted, by whom (child, parent or doctor) and for what reason; if any foods caused or worsened symptoms, if the child had any strong food dislikes and what they were, and finally, if any clinical GI diagnosis had ever been given. Parents/guardians answered each of the questions for both current (within the past three months) and previous experiences. Medications and/or behavioral therapies used at the time of enrollment or within the previous year were collected and recorded.

**Table 1:** Participant demographic information

	ASD-NoGI	ASD-GI	TD
N	24	12	18
Age, years (average)	7.40	8.10	7.54
Sex (F/M)	(5/19)	(3/9)	(2/16)
Autism Diagnostic Observation Schedule (mean, SD)			
Social Affect	13.8(4.1)	9(2.2)	-
Restrictive and Repetitive Behaviors	4(1.6)	3.3(1.1)	-
Severity Score	7.7(1.5)	6.2(1)	-
Autism Diagnostic Interview-Revised (mean, SD)			
Social Interaction	16.8(5)	16.8(4.4)	-
Verbal Communication	12.7(3)	16.9(3)	-
Non-Verbal Communication	10.4(2.1)	9.5(3.5)	-
Stereotyped Behaviors	6.6(2.6)	6.4(2.1)	-
Total Score	4.4(0.7)	3.9(0.9)	-
Mullen Scales of Early Learning, T-score (mean, SD)			
Visual Reception	36.6(16.1)	43.2(13.7)	55.3(12.3)
Fine Motor	34.5(14.8)	36.8(12.9)	57(15.8)
Receptive Language	29.5(11.2)	34.3(12.5)	51.1(10.2)
Expressive Language	28.7(9.9)	34.9(8.8)	49.6(12.4)
Early Learning Composite	70(19.3)	77.6(19.1)	106.5(19.8)
Aberrant Behavior Checklist (mean, SD)			
Irritability	14(9)	10(8)	4(4)
Lethargy	11(8)	8(7)	3(4)
Stereotypy	5(4)	4(4)	2(2)
Hyperactivity	10(9)	7(6)	3(4)
Inappropriate Speech	2(2)	2(2)	1(1)
Current CHARGE GI History (median, IQR)			
Abdominal Pain	0(1)	1(1)	0(0.25)
Gas/Bloating	0(1)	1(1)	0(0.25)
Diarrhea	0(1)	0.5(1)	0(1)
Constipation	0(1)	1(1)	0(0)
Pain on Stooling	0(0.25)	1(1.25)	0(0)
Vomiting	0(0)	0(0)	0(0)
Sensitivity to Foods	0(0.25)	0(1.25)	0(0)
Difficulty Swallowing	0(0)	0(0)	0(0)
Blood in Stool	0(0)	0(0)	0(0)
Blood in Vomit	0(0)	0(0)	0(0)

Participants were excluded if they had a known diagnosis of other GI pathology (celiac disease or inflammatory bowel diseases), use of antibiotics or antifungal medications within the prior month, medications affecting GI transit (stool softeners), and/or recent evidence of a GI infection based on stool laboratory tests performed by the child’s physician. In addition, participants were excluded if there was evidence of a seizure disorder, genetic disorders (i.e. Fragile X syndrome, Tuberous Sclerosis Complex), liver or pancreatic disease, cystic fibrosis, or chronic infection. Children receiving nutritional monitoring and prescribed dietary interventions under the guidance of trained nutrionists/clinicians, medications, or complementary alternative treatments such as supplements other than a standard daily multivitamin/mineral tablet were also excluded. However, for children who were receiving nutritional modifications that were not overseen by a trained nutritionist, the dietary changes were documented but the participants were not excluded from the study. Similarly, children whose parents reported suspected food sensitivities/intolerances that had not been diagnosed through clinical assessment were also not excluded, but were documented. Demographic and behavioral information can be found in Table 1. This study received approval from the institutional review boards at the University of California, Davis. Written and informed consent was obtained from the legal guardian or parent for all participants.

### Peripheral blood mononuclear cell (PBMC) and T cell isolation

Blood was collected into citrate tubes from study participants and centrifuged at 2100 RPM. The plasma layer was removed, and blood was resuspended in 1X Hanks Balanced Saline Solution (HBSS) (Corning, CA# 21-021-CM). Blood was then carefully layered onto Lymphoprep (Corning, CA# 25-072-CV) and centrifuged at 1700 RPM for 30 min. The PBMC layer was removed and washed with 40mL of 1X HBSS. PBMC were then resuspended in 1mL autoMACS Rinsing Solution (Miltenyi, CA# 130-091-222) supplemented with 0.05% BSA and filtered using a 40 μm filter into a new tube. PBMC were then washed with 10mL of autoMACS Rinsing Solution twice at 300 x g to remove contaminating platelets. An aliquot of PBMC was kept for analysis. Following manufacturer’s instructions, PBMC were enriched for CD4^+^CD25^+^ Tregs cells using Miltenyi CD4^+^CD25^+^ Regulatory T Cell Isolation kit, human (CA# 130-091-301). Purity of Tregs was performed by flow cytometry in samples where cells were enough for all assays and was 99 ± 1%. Cells derived from this isolation process were then used for flow cytometry and RNA sequencing.

### Cellular activation and flow cytometry

PBMC and T cells were rested or activated using CD3/CD28 conjugated beads provided by the Miltenyi T cell activation/expansion human kit. (CA# 130-091-441). 24 h post activation, cells were stained for flow cytometry analysis. Viability was accessed by staining with the Zombie Aqua Fixable Viability kid (BioLegend, CA# 423101) for 15 min. Cells were then washed twice with PBMC wash solution (1% BSA, 1% sodium azide), followed by incubation with Human TrueStain FcX (BioLegend CA# 422302) for 5 min. Without removing the Fc block, the following antibodies were added: CD3- BV605(clone UCHT1), CD4-Alexa Fluor 700 (clone RPA-T4) ,CD25-PE, Foxp3-Alexa 488(clone 206D), CD127-APC Fire 750(clone A019D5), a4b7-PercP-Cy5.5(clone FIB504), and BV421 conjugated: CCR9(clone L053E8) or CD39(clone A1) or Glycoprotein A repetitions predominant (GARP)(clone 7B11) or (CTLA-4 (clone BNI3) in combination with APC conjugated: TIGIT(clone A15153G) or Glucocorticoid-induced Tumor necrosis factor receptor-related protein (GITR) (clone 108 − 17) or latency-associated peptide (LAP) (clone TW4-2F8)-APC. All antibodies were acquired though BioLegend. Data was read on a BD LSR 2 and data was analyzed and visualized using FlowJo v9 software. Gating strategy for Tregs identification can be found in Supplemental Fig. 1.

### Tregs isolation, RNA extraction and sequencing

In a subset of subjects, CD4^+^CD25^+^Tregs were isolated for RNA sequencing (RNAseq) analysis (ASD = 13, 2 female; TD = 9, 1 female). After isolation, Tregs were flash frozen and stored at -80°C until analysis. RNA was isolated from stored samples using the Zymo Research Quick DNA/RNA Miniprep Plus extraction kit (CA# D7003). Flash frozen cells were resuspended in Proteinase K supplemented DNA/RNA Shield and incubated at room temperature for 30 minutes. Samples were then centrifuged and the RNA containing supernatant was removed and resuspended in DNA/RNA Lysis buffer at a 1:1 ratio, before being transferred to spin-away filters and centrifuged at 10,000 g for 30 seconds. A 1:1 volume of 95% ethanol was added to the flow through and transferred to a Zymo-Spin IIICG Column and centrifuged. Column contents were treated with DNase I for 15 minutes, followed by a wash with DNA/RNA Prep-Buffer. Columns were then washed twice with DNA/RNA Wash buffer and resuspended in 30µl of DNase/RNase-Free Water. RNA quality was assessed an Aligent Bioanalyzer. Samples with a DV200 score greater than 30% were submitted for library preparation. Libraries were prepared for 3’ Tag-Sequencing (RNAseq) (Lexogen) using 20ng of input RNA. Single end 80 bp reads were sequenced on an Aviti sequencer.

### Statistical analysis

Flow cytometry data passed normality tests; therefore, parametric tests were used for downstream analysis. Outlier removal was performed using ROUT, with Q = 1%. Unpaired Student’s t tests were used for between group comparisons. Spearman correlations were determined to be more appropriate for the present study, as this test is better adapted for the variability that comes with small sample sizes [33, 34]. Correlations between cell frequency, behaviors, and GI symptoms were generated. *P*-values were then corrected for multiple comparisons using the false discovery rate (FDR). FDR corrected *p*-values < 0.05 were considered statistically significant. Analysis and visualization of flow cytometry data was performed using GraphPad v9 and the SAS program JMP v16.

RNAseq Statistical Analysis data analysis and visualization was performed using the Partek Flow Software (Illumina). Raw data with Phred 33^+^ scores greater than 25 were aligned using the Spliced Transcripts Alignment to a Reference (STAR) local aligner. Samples with less than 50% alignment were removed from analysis. Aligned reads were quantified with an annotation model and the resulting gene counts were used for statistical analysis. Features with counts less than 1 were filtered prior to DESeq2 median ration normalization. Differential gene expression (DEGs) statistical analysis was performed using DESeq2 using Diagnosis and GI status as model parameters. Multiple testing was corrected for using false discovery rate (FDR). Genes with an FDR corrected *p*-value < 0.05 were considered significant. DEGs were analyzed and visualized for enriched Gene Ontology (GO) terms and Kyoto Encyclopedia of Genes and Genomes (KEGG) pathways using the online resource Metascape [35]. Multiple testing for GO and KEGG terms was corrected for using Benjamini Hochberg (BH) tests. BH adjusted *P* values < 0.05 were considered significant.

## Results

### Tregs frequencies are lower in ASD children with GI co-morbidities

Due to the low frequency of GI symptoms in TD children, and the lack of stability of symptoms over 2 timepoints we were unsuccessful at recruiting TD^GI^ children for the assays [10, 13]. However, in previous studies, we found TD^GI^ and TD^NoGI^ to be comparable for immune assays [16]. Due to concerns of age and sample size we choose to focus Tregs comparisons of the ASD^GI^ and ASD^NoGI^ groups when compared to the TD^NoGI^ group.

**Fig. 1:**
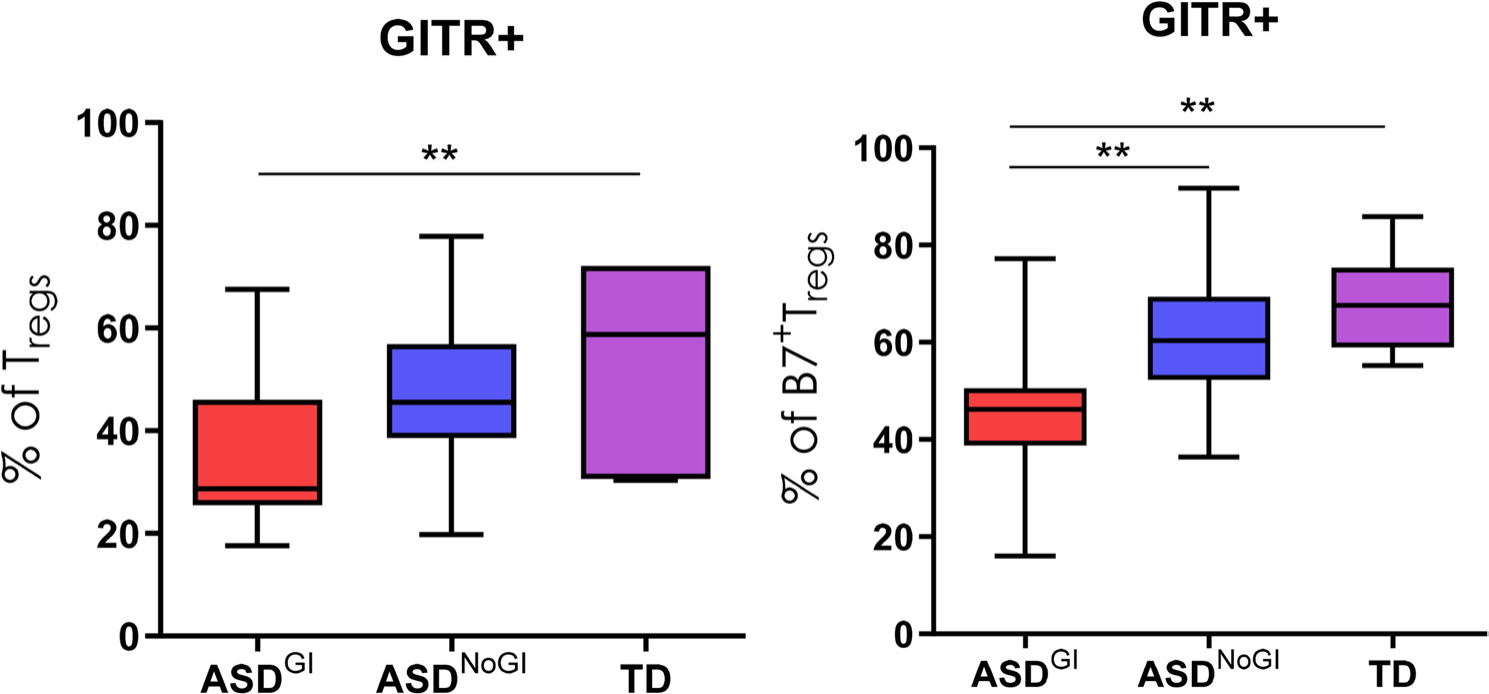
GITR expression on Tregs from ASD children with or without GI symptoms. Flow cytometry was used to access GITR expression on non-gut homing (left) and gut-homing α4β7 Tregs (right) from ASD^GI^, ASD^NoGI^, and TD kids. Both non-gut homing and gut-homing GITR expressing Tregs were significantly lower in ASD^GI^ children compared to TD controls. In gut-homing GITR^+^ Tregs, ASD^GI^ children had significantly lower frequencies than ASD^NoGI^ children also. Significance was determined as *p* < 0.05 (** = *p*-value < 0.005)

**Fig. 2:**
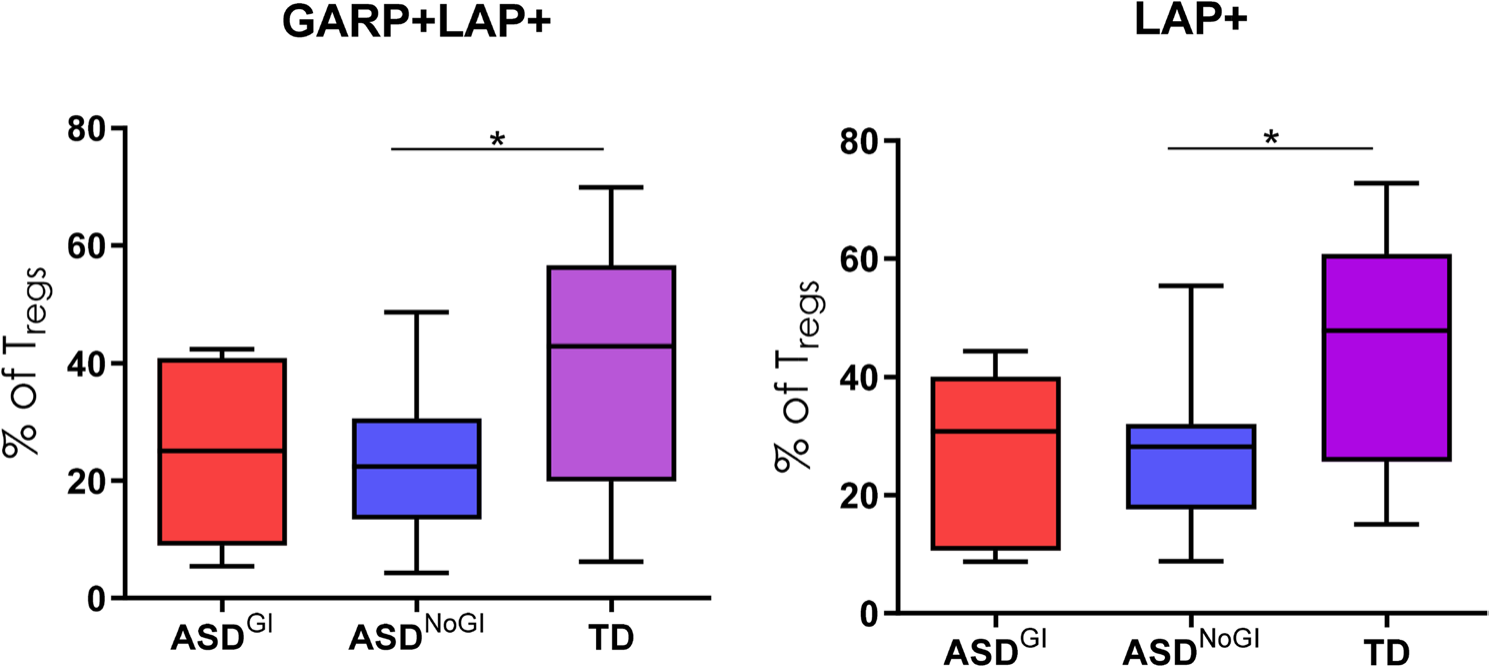
GARP and LAP expression on Tregs in ASD children with or without GI symptoms. Flow cytometry was used to access GARP and LAP expression on Tregs from ASD^GI^, ASD^NoGI^, and TD children. Tregs expressing LAP, as well as those co-expressing GARP and LAP surface markers, were significantly lower in ASD^NoGI^ children compared to TD controls. Significance was determined as *p* < 0.05 (*)

We have previously identified distinct immune profiles in ASD subjects based on the presence of GI issues [16, 20, 36]. We were unable to detect statistically significant differences in TIGIT and CTLA-4 Tregs among the groups. CD39^+^ Tregs tended to be reduced in ASD^GI^ compared to TD controls but did not reach statistical significance after correction for multiple comparisons (Tables 2, 3 and 4). When analyzing CD3^+^CD4^+^ that were positive for the gut homing molecule α4β7, ASD^GI^ subjects had lower frequencies of α4β7^+^CD127^−^CD25^+^Foxp3^+^ Tregs compared to ASD^NoGI^ subjects (ASD^GI^ mean = 6.1 ± 1.2; ASD^NoGI^ mean = 7.8 ± 2.5, *p* = 0.017). ASD^GI^ subjects also had fewer activated GITR^+^CD127^−^CD25^+^Foxp3^+^ Tregs compared to TD controls (ASD^GI^ mean = 34 ± 14.5; TD mean = 54.8, SD = 18.1, *p* = 0.008) with a trend towards lower frequencies compared to ASD^NoGI^ subjects (mean = 47 ± 16.4, *p* = 0.059) but this did not reach statistical significance (Fig. 1; Table 4). In addition, ASD^GI^ subjects exhibited lower α4β7^+^GITR^+^CD127^−^CD25^+^Foxp3^+^ Tregs (ASD^GI^ mean = 44 ± 15; TD mean = 67.9 ± 10, *p* = 0.006 compared to the TD and ASD^NoGI^ (ASD^NoGI^ mean = 60.8 ± 13, *p* = 0.001) (Fig. 1; Table 4). Conversely, there were decreased activated LAP^+^CD127^−^CD25^+^Foxp3^+^ Tregs in ASD^NoGI^ (ASD^NoGI^ mean = 29.6 ± 13.6; TD mean = 45.5 ± 20.2, *p* = 0.04) compared to TD controls and were similar to the ASD^GI^ group (mean = 28 ± 13.8)(Fig. 2). ASD^NoGI^ also had a decrease in GARP^+^ LAP^+^CD127^−^CD25^+^Foxp3^+^ Tregs compared to TD controls (ASD^NoGI^ mean = 23.7 ± 13.5, TD mean = 39.7 ± 22.2, *p* = 0.05), and again were comparable to ASD^GI^ subjects (mean = 24.6 ± 14.1) (Fig. 2). Similar findings also held true for α4β7^+^GARP^+^ CD127^−^CD25^+^Foxp3^+^ Tregs, which were decreased in ASD^NoGI^ compared to controls (ASD^NoGI^ mean = 38.8 ± 15.4, TD mean = 53.5 ± 13.3 *p* = 0.046), but not ASD^GI^ subjects (mean = 42.5 ± 19.6). In summary, our data suggested differences in Tregs in ASD compared to TD controls with differences in GITR staining dependent on GI status.

**Table 2:** CD3^+^CD4^+^ Peripheral Blood Mononuclear Cell Frequencies in children with ASD with or without GI symptoms [mean (SD)]

	**TD**	**ASD-NoGI**	**ASD-GI**
Foxp3^+^ PBMCs	6.4(1.8)	6.3(1.9)	6.6(1.6)
α4β7+ PBMCs	4.7(1.9)	3.1(1.5)*	4.6(1.2)^
α4β7^+^Foxp3^+^ PBMCs	4.7(1.6)	4.4(3.4)	5.1(2.5)
α4β7^+^CCR9^+^Foxp3^+^ PBMCs	1.9(0.8)	1.8(1.9)	1.7(0.8)
CCR9^+^ PBMCs	7.9(1.4)	7.8(2.8)	7.9(1.9)

* = compared to TD-NGI group, *p* < 0.05

^ = compared to ASD-NGI group, *p* < 0.05

**Table 3:** CD127^-^CD25^+^Foxp3^+^ (Tregs) Regulatory T cell Frequencies in children with ASD with or without GI symptoms [mean(SD)]

	TD	ASD-NoGI	ASD-GI
CD127^−^CD25^+^FoxP3^+^	4.5(1.35)	4.8(1.56)	4.6(2.10)
CD39^+^ Tregs	51.7(29.6)	33.3(37)	20(14.8)
α4β7^+^CD39^+^ Tregs	35.6(20.2)	31(21)	38.1(13.5)
TIGIT^+^ Tregs	44.8(6.7)	47.2(12.3)	50.8(8.2)
α4β7^+^TIGIT^+^ Tregs	54.9(7.9)	55.4(14.3)	59.5(15.5)
CD39^+^TIGIT^+^ Tregs	36.7(22.2)	31.7(37)	12.3(6.7)

**Table 4:** GITR, LAP, GARP in gut homing regulatory T cell (CD127^-^CD25^+^Foxp3^+^ Tregs) frequencies in children with ASD with or without GI symptoms [mean(SD)]

		**TD**	**ASD-NoGI**		**ASD-GI**
α4β7^+^ Tregs		7.3(4.1)	7.8(2.5)		6.1(1.2)^
CTLA-4^+^ Tregs		12.2(6.6)	12.9(6.8)		16.5(15)
α4β7^+^ CTLA-4^+^Tregs		14.3(8.6)	15.2(5.8)		18.7(16.8)
GITR^+^Tregs		54.8(18.1)	47(16.4)		34(14.5)*^
α4β7^+^ GITR^+^ Tregs		67.9(10)	60.8(13)		45(15)^*
GARP^+^ Tregs		50(19.4)	35.7(14.3)		39.8(16.1)
α4β7^+^ GARP^+^ Tregs		53.5(13.3)	38.8(15.4)*		42.5(19.6)
LAP^+^ Tregs		45.5(20.2)	29.6(13.6)*		28(13.8)
α4β7^+^ LAP^+^ Tregs		42.6(22.7)	32.1(17.8)		32.2(15)
GARP^+^LAP^+^ Tregs		39.7(22.2)	23.7(13.5)*		24.6(14.1)

^ = compared to ASD-NoGI group, *p* < 0.05

* = compared to TD group, *p* < 0.05

### The RNA transcriptome in isolated Tregs from children with ASD is enriched in genes related to chromatin organization and metabolism

**Fig. 3:**
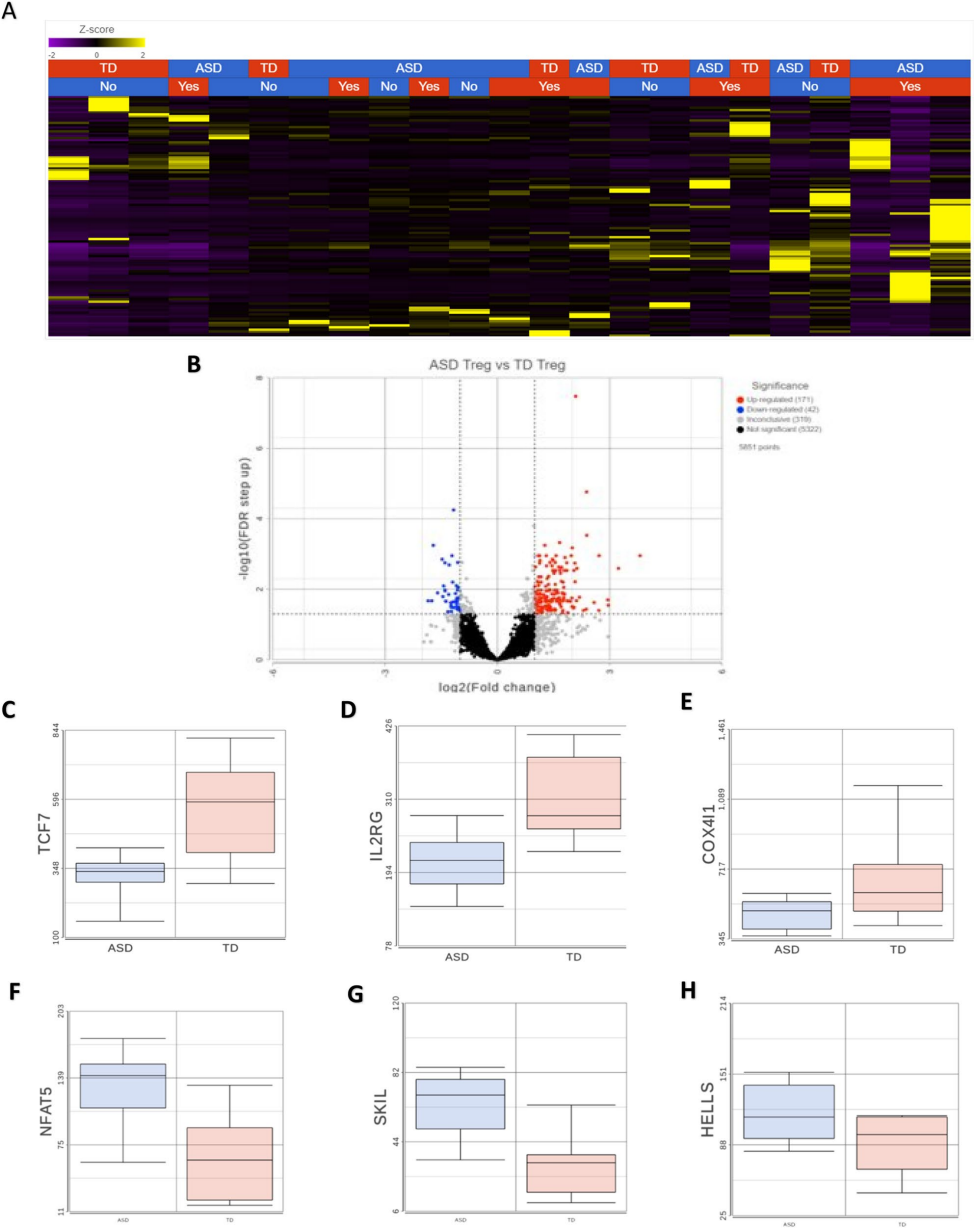
Transcriptional differences in Tregs from ASD and TD children. Bulk RNAseq was used to probe for transcriptional differences between ASD and TD Tregs. **A** Unsupervised hierarchical clustering was used to determine similar groups of subjects, with the first level being diagnosis and second being the presence (“Yes”) or absence (“No) of GI symptoms. Gene expression (rows) is represented by the Z score. **B** A Volcano Plot displaying differentially expressed genes that pass the *p*-value adjustment threshold. A total of 213 genes were identified as differentially expressed. **C – E** Individual downregulated genes *TCF7* (C), *ILRRG* (D) and *COX41* (E) between diagnostic conditions. **F – H** Individual upregulated genes *NFAT5* (F), *SKIL* (G) and *HELLS* (H) between diagnosis conditions

**Fig. 4:**
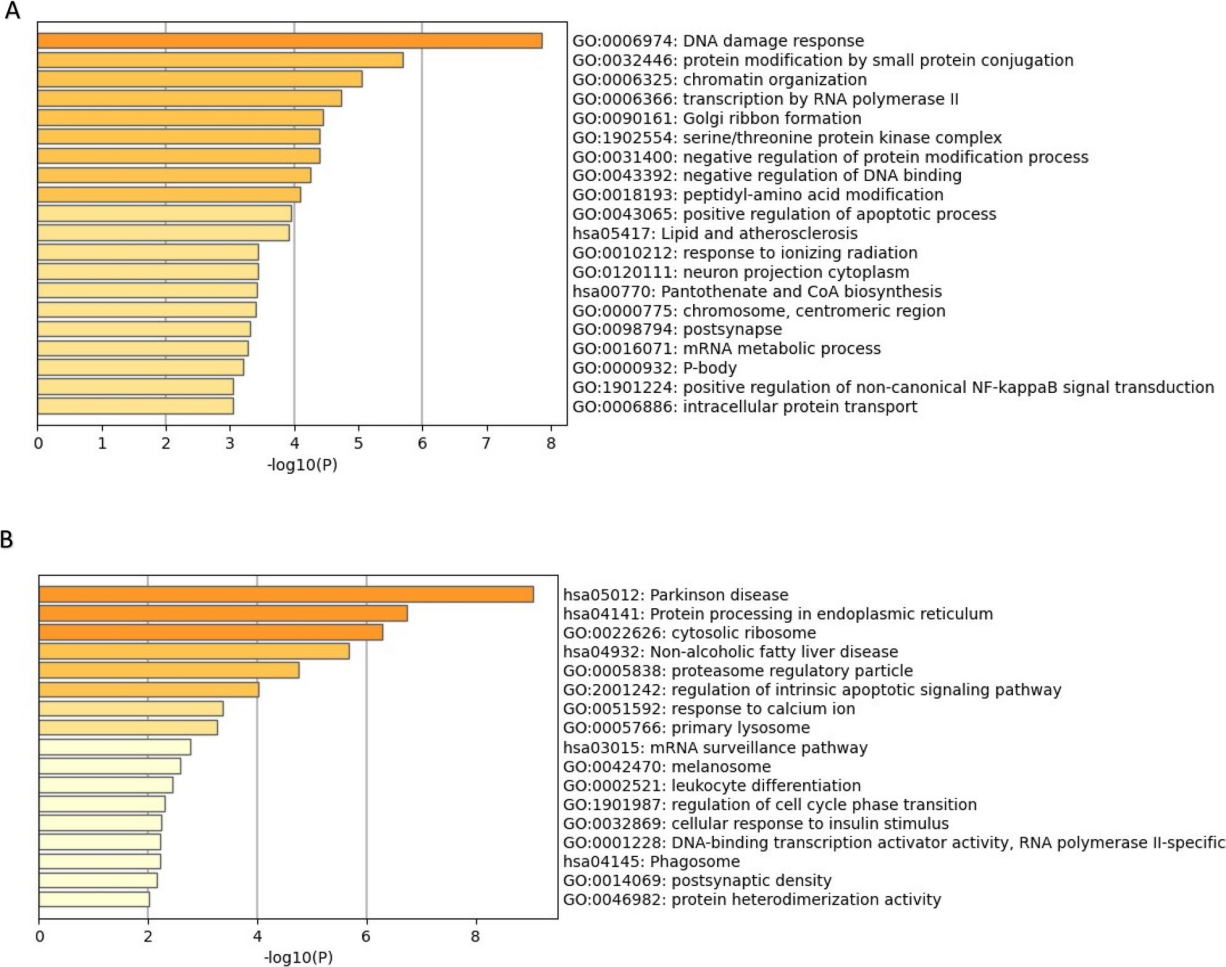
Differentially expressed Tregs genes in ASD are enriched in DNA remolding and metabolic pathways. Gene Ontology (GO) and Kyoto Encyclopedia of Genes and Genomes (KEGG) term enrichment analysis of upregulated (A) and downregulated (B) genes. **A** Upregulated DEGs from ASD Tregs were enriched in multiple GO terms related to DNA remolding, including ‘DNA damage response’ (GO:0006974), and ‘chromatin organization’(GO: 0006325). Significant KEGG pathways included ‘lipid and atherosclerosis’ (hsa05417) and ‘Pantothenate and CoA biosynthesis’ (hsa00770). **B** Downregulated DEGs from ASD Tregs were enriched pathways related to metabolism, including ‘Parkinson disease’ (hsa05012), as well as immune function (‘leukocyte differentiation’) and protein biology (‘Protein processing in endoplasmic reticulum’)

Unsupervised clustering revealed transcriptional differences between ASD and TD groups (Fig. 3A). We identified 213 DEGs when comparing Tregs from children with ASD to TD controls, of which 171 were upregulated and 42 downregulated (Fig. 3B). Upregulated genes were enriched in several GO terms related to DNA remodeling, including ‘DNA damage response’, chromatin organization’ and ‘negative regulation of DNA binding’ (Fig. 3B). These terms consisted of genes such as the helicase, lymphoid specific (HELLS) (Fig. 3H) (involved in methylation activity), enhancer of zest 1 polycomb repressive complex 2 (EZH1) (involved in the methylation of histones), and the transcription activation suppressor (TASOR) gene (enables chromatin binding). Furthermore, upregulated DEGs were enriched in terms and pathways related to metabolism, including the KEGG pathway ‘lipid and atherosclerosis’, including genes such as low-density lipoprotein receptor (LDLR) and POU Class 2 Homeobox 1 (POU2F1), and the ‘Panthothenate and CoA biosynthesis’ pathway, including the genes aldehyde dehydrogenase 3 family member A2 (ALDH3A2) and pantothenate kinase 3 (PANK3) (Fig. 4A). There were several upregulated genes associated with immune signaling, such as mitogen-activated protein kinase 3 (MAPK3), janus kinase 2 (JAK2), nuclear factor of activated T cells 5 (NFAT5) (Fig. 3F), SKI like proto-oncogene (SKIL) (Fig. 4) and zinc finger MIZ-type containing 1 (ZMIZ1). For the 43 downregulated genes, these were enriched for terms related to processes involved in oxidative phosphorylation, such as the GO Biological process terms ‘cellular respiration’ and ‘oxidative phosphorylation’ (Fig. 4B, Supplementary File 2). In addition, other terms related to immune function, including ‘MAPK signaling pathway’ and ‘leukocyte differentiation’, and included the transcription factor 7 (TCF7) (Fig. 3C) and the interleukin 2 receptor subunit gamma (IL2RG) gene (Fig. 3D, Supplementary File 2). KEGG pathways were associated with protein processing and protein localization, such as the ‘protein processing in endoplasmic reticulum’ pathways (Supplementary File 2). An enrichment of terms related to the cellular stress response was found, including the GO term ‘regulation of intrinsic apoptotic signaling pathway’, which included the genes endoplasmic reticulum to nucleus signaling 1 (ERN1), selenoprotein S (SELENOS), transcription factor AP4 (TFAP4) and cell cycle and apoptosis regulator 2 (CCAR2). These data suggest that there are dysregulated immune, metabolic and DNA remodeling programs in Tregs from ASD, in addition to an altered stress state.

Next, in preliminary analysis, we considered how GI status may influence DEG enrichment. Comparison of mRNA data from ASD^GI^ and TD Tregs reveal a total of 19 DEGs, with 4 upregulated and 15 downregulated (Supplemental File 1). Upregulated genes were associated with the meiotic cell cycle (PDIK1L) and tumor proliferation (BACH1 and PDIK1L). Within the downregulated genes, several were involved in mitochondrial function (MT-ND1, MT-CYB, MT-ATP6, and CYB5R1). In ASD^NoGI^ and TD comparisons, only 7 DEGs were observed (5 downregulated/2 upregulated) after multiple comparison adjustment (Supplemental File 1). Upregulated genes consisted of MIB2 and SLC39A10 and did not seem to be involved in any pathway. Downregulated genes included DPP7, CIRBP, MT-ND1, RBM3 and TRAPPC6A and also did not appear to be involved in any molecular, cellular or biological pathway.

### Fewer Tregs are associated with worse behaviors in TD and ASD children

**Fig. 5:**
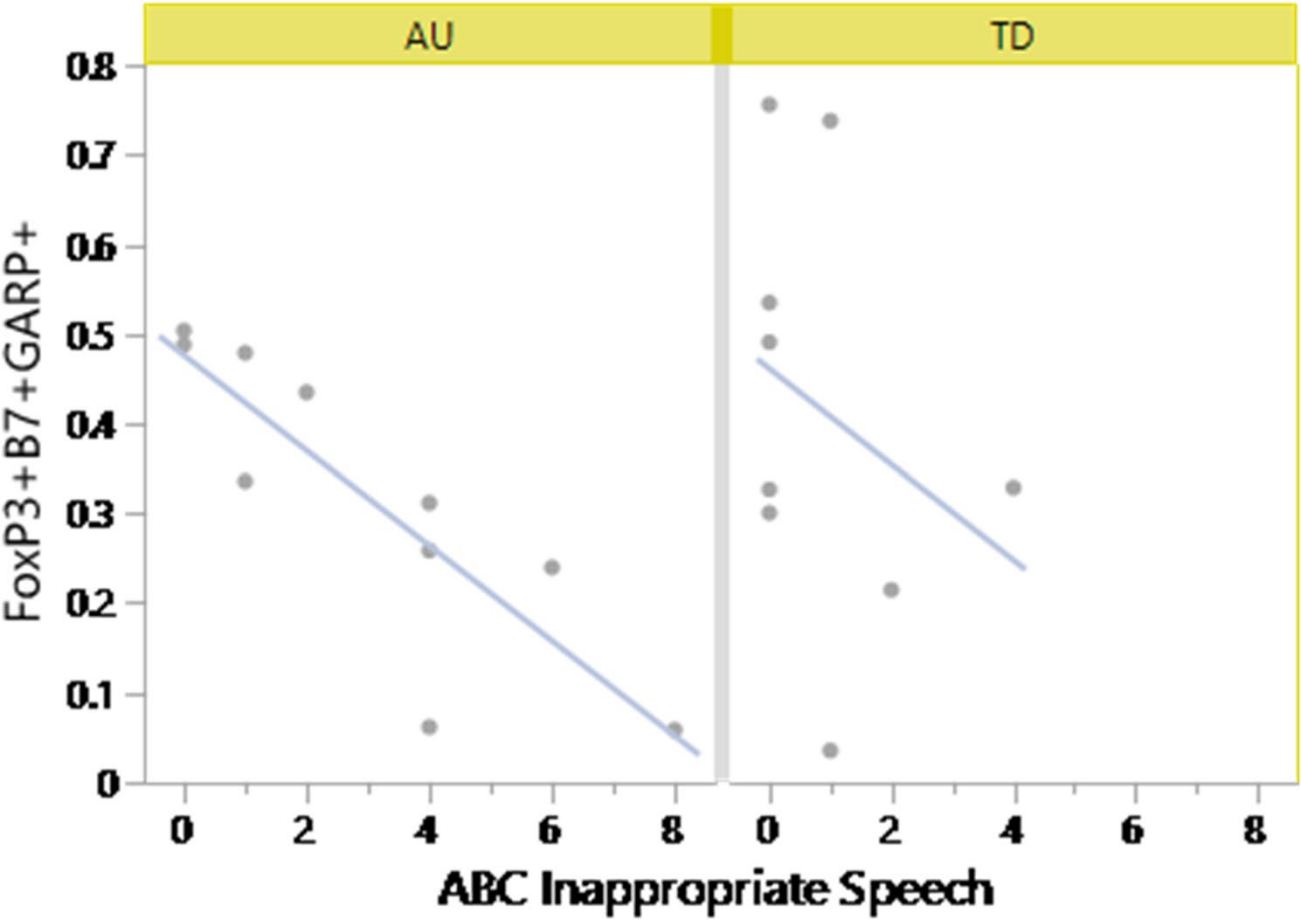
Gut homing GARP^+^CD127^−^CD25^+^Foxp3^+^ Tregs are associated with improved behaviors. Spearman Rho correlations were used to access associations between baseline and activated Tregs frequencies and ADI, MSEL, VABS and ABC assessment scores in ASD and TD subjects. Significant correlations were observed between gut-homing α4β7^+^GARP + CD127^−^CD25^+^Foxp3^+^ Tregs and Inappropriate Speech behavior within the ASD^NoGI^ group (rho = -0.9384, FDR *P*-value = 0.0021)

To gain further insights into the relationship between Tregs and ASD related behaviors, we investigated whether there were associations between behavioral scores, the GI assessments and the phenotypes of Tregs (Table 5). In the context of ASD, we identified a statistically significant association between increased α4β7^+^GARP^+^CD127^−^CD25^+^Foxp3^+^Tregs and improved ABC inappropriate speech scores in the ASD^NoGI^ group. (rho = -0.9384, FDR *p*-value = 0.0021) (Fig. 5). However, after multiple comparisons correction, no significant correlations were observed in the ASD^GI^ group alone or based on GIH assessment scores across the ASD groups. In the TD group, decreased Tregs were associated with lower adaptive behaviors, primarily between GARP^+^LAP^+^CD127^−^CD25^+^Foxp3^+^ and LAP^+^CD127^−^CD25^+^Foxp3^+^ Tregs, which were associated with reduced VABS composite (rho = -0.833, FDR *p*-value = 0.016) and daily living scores (rho = -0.7833, FDR *p*-value = 0.037), respectively.

**Table 5:** Tregs cell phenotype and associations with behaviors

Group	Cell	Behavior	Spearman	FDR *P* -Value
ASD-NoGI				
	Foxp3^+^α4β7^+^GARP^+^	ABC Inappropriate Speech	-0.9384	0.0021
TD				
	Foxp3^+^α4β7^+^	MSEL Visual Reception	0.8536	0.011
	Foxp3^+^α4β7^+^CD39^+^	Vine socialization	0.8488	0.012
	Foxp3^+^Lap^+^	Vine Composite	-0.833	0.016
	Foxp3^+^Garp + LAP^+^	Vine Daily Living	-0.7833	0.037
	Foxp3^+^α4β7^+^GARP^+^LAP^+^	Vine Communication	0.7833	0.037
	CD3^+^CD4^+^	MSEL Visual Reception	0.7078	0.044
	Foxp3^+^α4β7^+^CD39^+^TIGIT^+^	Vine socialization	0.7637	0.049

## Discussion

Immune dysregulation is a common feature observed in children with ASD. Immune suppression via Tregs is essential for maintaining homeostasis. Disruptions to regulatory mechanisms can result in inflammation and an increased likelihood of developing immune related conditions. In the current study, we sought to characterize Tregs in ASD and whether differences in Tregs are more apparent in children with ASD and stable gastrointestinal comorbidities. We found that ASD^GI^ children had reduced GITR^+^CD127^−^CD25^+^Foxp3^+^Tregs compared to TD controls, and reduced gut homing α4β7^+^GITR^+^CD127^−^CD25^+^Foxp3^+^Tregs compared to ASD^NoGI^ and TD controls. Furthermore, we observed lower LAP^+^CD127^−^CD25^+^Foxp3^+^Tregs, GARP^+^ LAP^+^CD127^−^CD25^+^Foxp3^+^ Tregs, and gut homing α4β7^+^GARP^+^CD127^−^CD25^+^Foxp3^+^ Tregs in ASD^NoGI^ compared to their TD counterparts. Analysis of gene expression in isolated CD4^+^CD25^+^Tregs revealed that upregulated genes in children with ASD were primarily involved in DNA remodeling/modification, metabolism and immune signaling. Downregulated genes in ASD belonged to processes involved in oxidative phosphorylation, as well as genes involved in T cell differentiation and protein biology. Finally, we found some evidence that suggested there were associations between worsening behaviors and decreased Tregs both in the context of ASD and TD status, consistent with previous studies on immunosuppressive cytokines [22]. Restoring Tregs function may thus prove to be a beneficial strategy for ameliorating potentially harmful immune activation in the context of ASD.

Inflammatory conditions in peripheral, central nervous system, and GI tissues are frequently observed in ASD. Increased concentrations of peripheral pro-inflammatory cytokines IL-1β, IL-6, IL-8 and IL-12p40 are seen in ASD and are associated with more impaired behaviors [9]. Elevated levels of TNFα, IL-6, IFNγ, GM-CSF and IL-8 are also observed in the post-mortem brain tissue of ASD children [37]. Evidence of inflammatory cells have also been found in gastrointestinal tissues [38]. These findings may in part be due to the increased frequencies of activated immune cells in each of these respective tissues [19, 24, 39, 40]. However, another contributing factor to this seemingly systematic inflammation is dysregulated Tregs biology. In ASD, lower frequencies of peripheral Tregs and the canonical Tregs-derived cytokines TGFβ, IL-10 and IL-35 are reported [23, 41, 42]. Deficiencies in putative Tregs were first suggested by Warren et al., over 35 years ago, who identified lower peripheral frequencies of CD4^+^CD45RA^+^ T cells [43]. Ahmed and colleagues found that transcription factors associated with a Tregs phenotypic identity, primarily Foxp3 and Helios, are reduced in ASD; however, few studies have attempted to identify different Treg phenotypes in ASD [44, 45]. Using CD127^−^CD25^+^Foxp3^+^ as the basis to identify Tregs, the results of the current study showed that various Tregs phenotypes were lower in children with ASD. The frequencies of Tregs positive for the anti-inflammatory molecules LAP^+^, GARP^+^, and GITR^+^ were different in ASD compared to controls, varying based on the presence of GI symptoms.

The immune modulatory marker LAP is non-covalently associated with active TGFβ1, rendering TGFβ1 biologically inactive until cleaved [46]. GARP binds to LAP and upon tethering to the Tregs surface, TGFβ1 is converted to its activated form though integrins, resulting in the release of active TGFβ1 [46, 47]. In the present study, we found evidence of GARP and LAP dysregulation in ASD children. We observed lower frequencies of LAP^+^GARP^+^CD127^−^CD25^+^Foxp3^+^ and LAP^+^CD127^−^CD25^+^Foxp3^+^ Tregs, as well as reduced gut homing α4β7^+^GARP^+^CD127^−^CD25^+^ Foxp3^+^ Tregs. There are several possible reasons as to why there are decreased GARP^+^ and LAP^+^ Tregs, such as genetic mutations, inefficient Tregs activation, or dysregulated membrane trafficking [48, 49]. For example, in a study by Lehmkuhl et al., investigating patients with primary immunodeficiencies, mutations in the GARP gene, LRRC32, was associated with severely reduced Tregs populations and reduced suppressive activity [48]. Furthermore, reduced GARP expression could contribute a destabilized Tregs phenotype. Mechanistically, this may result from GARP mediated regulation of the histone deacetylase HDAC9. Deficiencies in GARP increased Hdac9 activity and resulted in deacetylation of Foxp3 (the Tregs “master controller”) at a greater rate, leaving Foxp3 more susceptible to removal from the cellular system [48]. Reduced LAP may be a consequence of reduced GARP expression, as the latter tethers the former to the cell surface [49]. In this study we found evidence that suggested reduced gut-homing GARP^+^ Tregs to be associated with worsening inappropriate speech scores. Although these associations are interesting, it is noted that few statistically significant associations were found between behavioral assessments and Tregs phenotypes in the ASD groups after correcting for multiple comparisons and thus we treat these data with caution. There were more associations between behaviors and reduced Tregs frequencies in the context of the TD group. Future confirmatory experiments focused on Tregs phenotypes and behavior in independent populations are warranted. 

GITR is a member of the TNF-receptor superfamily and binds to its ligand, GITRL, typically found on antigen presenting and endothelial cells [50, 51]. Tregs express high levels of GITR on their membranes, where it acts as a co-stimulatory molecule [52]. GITR^+^ Tregs are markedly lower in frequency in several autoimmune diseases including type 1 diabetes and systemic lupus erythematous. Increased frequency of GITR^+^ Tregs is associated with the clinical remission in these conditions [53–56]. The role of GITR signaling in Tregs is nuanced, with data supporting GITR signaling in Tregs proliferation, expansion and prevention of inflammatory colitis in mice, while others have suggested that during GITR mediated proliferation suppressive ability is abrogated. In the present study, we observed reduced frequencies of GITR^+^CD127^−^CD25^+^ Foxp3^+^Tregs in ASD^GI^ children. The function and significance of GITR^+^ Tregs in ASD children is unknown, although it can be speculated that reduced GITR^+^ Tregs may lead to GI related issues as shown in mice models [55, 56].

To the best of our knowledge, the RNA transcriptome of isolated Tregs from ASD children has not been investigated. In the present study, we identified several biological pathways that are crucial for Tregs mediated suppression and cellular identity. Firstly, many of the observed upregulated DEGs were involved in DNA remolding, such as HELLS and EZH1, both involved in DNA methylation. Significantly enriched GO terms included ‘chromatin organization’, consisting of genes involved in transcriptional regulation (Transcription Activation Suppressor; TASOR, Tousled-like kinase 1; TSK1, Myb Like, SWIRM and MPN Domains 1; MYSM1) as well as genes associated with histone deacetylases (ring finger protein, LIM domain interacting; RLIM, ligand dependent nuclear receptor interacting factor 1; LRIF1) [57–60]. Reorganization of chromatin structures and methylation at promoter regions of Tregs signature genes is essential for the development and maintenance of Tregs, with some subpopulations more resistant to changes in epigenetic status than others. Demethylation at the Treg-specific demethylated region (TSDR) within the Foxp3 locus is considered essential for Tregs development [61]. Upregulated expression of genes involved in epigenetic processes may indicate that epigenetic imprints at essential sites for Tregs phenotype stability are altered, potentially contributing to reduced Tregs in ASD. Moreover, genes that negatively regulate Foxp3 activity were upregulated, such as those involved in ubiquitination, including E3 ubiquitin ligases (HECT, UBA and WWE domain containing 1, E3 ubiquitin protein ligase; HUWE1, mind bomb 1; MIB1), and those that target ubiquitin tagged proteins for autophagy (sequestosome-1, SQSTM1). Since Foxp3 is negatively regulated by ubiquitination and acetylation, future investigation should focus on how much these protein modifications contribute to overall Tregs instability in neurodevelopmental disorders [62]. While both work to negatively regulate Foxp3 function, acetylation is reversable whereas ubiquitin tags molecules for permanent degradation. Overall, changes in the expression of genes involved in DNA remodeling machinery and protein modification could contribute to dysregulated Tregs phenotypes and consequently lower peripheral Tregs populations in ASD.

Dysregulated Tregs may also be due to altered cellular metabolic programs. Many of the metabolites and cofactors produced as a result of cellular metabolism modulate Tregs development and function [63]. Activated Tregs have increased glycolytic demands for energy and are tightly regulated to modulate Tregs phenotype and function [63]. For example, fatty acid metabolism and subsequent oxidative phosphorylation (OXPHOS) is associated with superior suppressive capabilities in Tregs [63]. In the present study, we identified several dysregulated metabolic genes. Upregulated genes were involved in aspects of lipid metabolism, including low-density lipoprotein receptor (LDLR), salt inducible kinase 2 (SIK2) – involved in fatty acid oxidation, the zinc finger DHHC-type palmitoyltransferase 21 (ZDHHC21) – a hormone receptor, and pantothenate kinase 3 (PANK3) gene - involved in Coenzyme A synthesis. Furthermore, GO terms associated with amino acid metabolism, such as ‘serine family amino acid metabolic process’ were found. Conversely, downregulated genes suggested disruptions in mitochondrial respiration and OXPHOS. Significantly disrupted GO Biological Process terms included ‘electron transfer activity’, ‘aerobic respiration’ and ‘oxidative phosphorylation’ (Supplemental data). Genes within these processes included those involved with mitochondrial cytochromes (cytochrome C oxidase subunit 4I1; COX4I1, cytochrome B; CYTB) and mitochondrial complex 1 (NADH dehydrogenase 1; ND1, NADH: upiquinone oxidoreductase subunit A10;NDUFA10). These data suggested that there are differences in Tregs metabolism between ASD and TD children, which could subsequently impact their phenotype and function. In ASD, we and others have shown that altered metabolism in PBMC and lymphoblast cell lines (mainly B cells) is present, suggesting altered metabolism could affect or be the consequence of increased immune responses [64, 65]. A recent single-cell RNA sequencing study evaluating the metabolic differences between T_H_17 cells and Tregs showed amino acid metabolism, specifically polyamine metabolism, was downregulated in Tregs [63]. Our studies suggest that ASD Tregs may preferentially undergo beta-oxidation and that genes involved with downstream tricarboxylic acid (TCA) and OXPHOS activity may be dysregulated.

There are several limitations to our study that require addressing. As FoxP3 is X linked, Tregs from females may have different phenotypes and gene expression compared to males. This study was not powered to determine differences in Tregs populations and gene expression changes between male and female groups based on diagnosis. Furthermore, due to the absence of a stable TD^GI^ group, we were unable to compare ASD^GI^ and TD^GI^ groups in our study. This may in part be due to the fact the study was focused on stable GI symptoms over two-time points rather than transient GI symptoms that could occur at any time point. GI symptoms had previously been measured in CHARGE, using the same assessment tool (GIH), and then evaluated at the second time point in this study. Only those participants with GI issues at both time points were considered part of the GI groups, and those without GI symptoms at both timepoints as part of the noGI groups. Recently we reported that GI symptoms are not only more frequent in ASD, with more symptoms described per ASD child but they are more stable occurring at multiple timepoints. In comparison GI symptoms in TD are much more transient, occurring at different time points but not with stability. Thus, making ASD^GI^ a potentially very different group than a transient GI symptom in similarly aged children [13]. This was an issue in prior studies due to the rarity or stability of GI issues in the TD population in this age group in the context of a population based cohort [16, 17]. Recruitment from specialized GI clinics has also substantial problems with symptoms recorded, diagnosis of a GI condition (that is absent in ASD^GI^ group), stability of symptoms and a lack of diagnostic testing for behaviors. To address these study population issues, collaboration across studies and institutes with access to a large pool of TD populations is necessary. Furthermore, a large variety of markers have been associated with a Tregs phenotype or suppressive ability. We focused on some of the better described surface molecules related to Tregs function due to the limited number of cells that could be collected from each pediatric blood sample. Further studies are warranted that expand the array of potential phenotypes assessed, potentially with methods less restrictive or biased as traditional flow cytometry. Due to the limited sample of Tregs in pediatric blood samples, we were also unable to confirm our RNAseq data with additional DNA modification and metabolomic assays. This prevented further investigation of Tregs specific dysregulation in ASD, as some of the KEGG and GO terms found have been broadly identified within the context of neurodevelopmental disorders, including previous studies from our lab [66]. Future studies on this topic are warranted. Lastly, we did not identify differences in the transcriptome of Tregs between ASD^NoGI^ and ASD^GI^ groups, which may be due to small sample sizes. Despite these limitations, we feel that the information provided in the present study significantly advances the fields understanding of Tregs dysregulation in ASD.

Tregs dysregulation may be at the root of many inflammatory processes that occur in ASD. In this study, we identified altered Tregs phenotypes in ASD, with some phenotypes being dependent on the presence of GI symptoms. We have also found that the gene pathways in Tregs are dysregulated in ASD. These pathways may affect DNA repair and cellular metabolism in ASD. Taken together, these data confirm the presence of Tregs dysregulation in ASD and provide a basis for future mechanistic and therapeutic studies. Indeed, recent pre-clinical targeting the increased generation of Tregs showed promising benefits towards alleviating some of the behavioral and physiological symptoms related to ASD [67]. Continued efforts are needed to fully understand how Tregs intersect with ASD and immune dysregulation.

## Supplementary Information

Supplementary Material 1.

Supplementary Material 2.

Supplementary Material 3.

## Data Availability

The datasets used for the current states are available from the corresponding author upon reasonable request.

## References

[1] Maenner MJ, et al. Prevalence and characteristics of autism spectrum disorder among children aged 8 Years — autism and developmental disabilities monitoring Network, 11 Sites, united States, 2020. MMWR Surveillance Summaries. 2023;72(2):1–14. 10.15585/mmwr.ss7202a1 PMC10042614 36952288

[2] Arenella M, et al. Potential role for immune-related genes in autism spectrum disorders: evidence from genome-wide association meta-analysis of autistic traits. Autism. 2021. 10.1177/13623613211019547. 10.1177/13623613211019547 PMC8814945 34344231

[3] Onore C, et al. Dynamic Akt/mTOR signaling in children with autism spectrum disorder. Front Pead. 2017;5:43. 10.3389/fped.2017.00043 PMC5350147 28361047

[4] Trifonova EA, et al. Do autism spectrum and autoimmune disorders share predisposition gene signature due to mTOR signaling pathway controlling expression? Int J Mol Sci. 2021;22(10):5248. 34065644 10.3390/ijms22105248 PMC8156237

[5] Tamayo JM, et al. Maternal allergic asthma induces prenatal neuroinflammation. Brain Sci. 2022;12(8):1041. 36009104 10.3390/brainsci12081041 PMC9405898

[6] Kim E, et al. Maternal gut bacteria drive intestinal inflammation in offspring with neurodevelopmental disorders by altering the chromatin landscape of CD4+ T cells. Immunity. 2021. 10.1016/j.immuni.2021.11.005 PMC8755621 34879222

[7] Patel S, et al. Maternal immune conditions are increased in males with autism spectrum disorders and are associated with behavioural and emotional but not cognitive co-morbidity. Translational Psychiatry. 2020;10(1):286. 32796821 10.1038/s41398-020-00976-2 PMC7429839

[8] Hughes HK, et al. Immune dysfunction and autoimmunity as pathological mechanisms in autism spectrum disorders. Front Cell Neurosci. 2018;12:405. 30483058 10.3389/fncel.2018.00405 PMC6242891

[9] Ashwood P, et al. Elevated plasma cytokines in autism spectrum disorders provide evidence of immune dysfunction and are associated with impaired behavioral outcome. Brain Behav Immun. 2011;25(1):40–5. 20705131 10.1016/j.bbi.2010.08.003 PMC2991432

[10] Chaidez V, Hansen RL, Hertz-Picciotto I. Gastrointestinal problems in children with Autism, developmental delays or typical development. J Autism Dev Disord. 2014;44(5):1117–27. 24193577 10.1007/s10803-013-1973-x PMC3981895

[11] Zerbo O, et al. Immune mediated conditions in autism spectrum disorders. Brain Behav Immun. 2015;46:232–6. 25681541 10.1016/j.bbi.2015.02.001 PMC4414798

[12] Hertz-Picciotto I, Croen LA, Hansen R, Jones CR, van de Water J, Pessah IN. The CHARGE study: an epidemiologic investigation of genetic and environmental factors contributing to autism. Environ Health Perspect. 2006;114(7):1119–25. 10.1289/ehp.8483. 16835068 10.1289/ehp.8483 PMC1513329

[13] Restrepo B, Taylor SL, Ponzini MD, Angkustsiri K, Solomon M, Rogers SJ, Ashwood P, Say DS, Caceres S, Alavynejad S, et al. A longitudinal evaluation of Gastrointestinal symptoms in children with autism spectrum disorder. Autism. 2025;29(11):2832–45. 10.1177/13623613251362349. 40877047 10.1177/13623613251362349 PMC12404668

[14] Moreno RJ, Amara RA, Ashwood P. Toward a better Understanding of T cell dysregulation in autism: an integrative review. Brain Behav Immun. 2025;123:1147–58. 39378971 10.1016/j.bbi.2024.10.009

[15] Ellul P, et al. Regulatory T lymphocytes/Th17 lymphocytes imbalance in autism spectrum disorders: evidence from a meta-analysis. Mol Autism. 2021;12(1):68. 34641964 10.1186/s13229-021-00472-4 PMC8507168

[16] Rose DR, et al. Differential immune responses and microbiota profiles in children with autism spectrum disorders and co-morbid Gastrointestinal symptoms. Brain Behav Immun. 2018;70:354–68. 29571898 10.1016/j.bbi.2018.03.025 PMC5953830

[17] Rose DR, et al. T cell populations in children with autism spectrum disorder and co-morbid Gastrointestinal symptoms. Behav Immun - Health. 2020;2:100042. Brain. 10.1016/j.bbih.2020.100042 PMC8474588 34589832

[18] Careaga M, et al. Immune endophenotypes in children with autism spectrum disorder. Biol Psychiatry. 2017;81(5):434–41. 26493496 10.1016/j.biopsych.2015.08.036 PMC4788581

[19] Hughes HK, R.J.Moreno, and, Ashwood P. Innate immune dysfunction and neuroinflammation in autism spectrum disorder (ASD). Brain Behav Immun. 2023;108:245–54. 36494048 10.1016/j.bbi.2022.12.001

[20] Moreno RJ, et al. Altered monocyte populations and activation marker expression in children with autism and Co-Occurring Gastrointestinal symptoms. Biomolecules. 2025;15(2):207. 40001509 10.3390/biom15020207 PMC11853397

[21] Dikiy S, Rudensky AY. Principles of regulatory T cell function. Immunity. 2023;56(2):240–55. 36792571 10.1016/j.immuni.2023.01.004

[22] Ashwood P, et al. Decreased transforming growth factor beta1 in autism: A potential link between immune dysregulation and impairment in clinical behavioral outcomes. J Neuroimmunol. 2008;204(1–2):149–53. 18762342 10.1016/j.jneuroim.2008.07.006 PMC2615583

[23] Rose D, Ashwood P. Rapid communication: plasma Interleukin-35 in children with autism. Brain Sci. 2019;9(7):152. 31252635 10.3390/brainsci9070152 PMC6680732

[24] Ashwood P, et al. Immune activation of peripheral blood and mucosal CD3+ lymphocyte cytokine profiles in children with autism and Gastrointestinal symptoms. J Neuroimmunol. 2006;173(1–2):126–34. 16494951 10.1016/j.jneuroim.2005.12.007

[25] Warren RP, et al. *Deficiency of Suppressor-Inducer (Cd4*^+^*Cd45ra+) T cells in autism*. Immunol Investig. 2009;19(3):245–51. 10.3109/08820139009041839 2142123

[26] Mostafa GA, Shehab AA, Fouad NR. *Frequency of CD4*^+^*CD25high regulatory T cells in the peripheral blood of Egyptian children with autism*. J Child Neurol. 2010;25(3):328–35. 19713552 10.1177/0883073809339393

[27] Nie Z-Q, et al. TH1/Treg ratio May be a marker of autism in children with immune dysfunction. Res Autism Spectr Disorders. 2023;101:102085.

[28] Alhosaini K, et al. Dysregulation of Ki-67 expression in T cells of children with autism spectrum disorder. Children. 2021;8(2):116. 33562037 10.3390/children8020116 PMC7915849

[29] Safari MR, et al. FOXP3 gene variations and susceptibility to autism: A case–control study. Gene. 2017;596:119–22. 27751813 10.1016/j.gene.2016.10.019

[30] Akbari M, et al. Assessment of expression of regulatory T cell differentiation genes in autism spectrum disorder. Front Mol Neurosci. 2022;15:939224. 35860502 10.3389/fnmol.2022.939224 PMC9289514

[31] Hughes HK, Ashwood P. Anti-Candida albicans IgG antibodies in children with autism spectrum disorders. Front Psychiatry. 2018;9:627. 30534090 10.3389/fpsyt.2018.00627 PMC6275220

[32] Ashwood P, Krakowiak P, Hertz-Picciotto I, Hansen R, Pessah IN, de Water JV. Altered T cell responses in children with autism. Brain Behav Immun. 2011;25(5):840–9. 10.1016/j.bbi.2010.09.002. 20833247 10.1016/j.bbi.2010.09.002 PMC3039713

[33] Bates B, et al. The effects of sample size and variability on the correlation coefficient. Med Sci Sports Exerc. 1996;28(3):386–91. 8776228 10.1097/00005768-199603000-00015

[34] Winter JCF, Gosling, Potter J. Comparing the pearson and spearman correlation coefficients across distributions and sample sizes: A tutorial using simulations and empirical data. Psychol Methods. 2016;21(3):273–90. 27213982 10.1037/met0000079

[35] Zhou Y, et al. Metascape provides a biologist-oriented resource for the analysis of systems-level datasets. Nat Commun. 2019;10(1):1523. 30944313 10.1038/s41467-019-09234-6 PMC6447622

[36] Ashwood P. Preliminary findings of elevated inflammatory plasma cytokines in children with autism who have Co-Morbid Gastrointestinal symptoms. Biomedicines. 2023;11(2):436. 36830973 10.3390/biomedicines11020436 PMC9952966

[37] Li X, et al. Elevated immune response in the brain of autistic patients. J Neuroimmunol. 2009;207(1–2):111–6. 19157572 10.1016/j.jneuroim.2008.12.002 PMC2770268

[38] Ashwood P, et al. Spontaneous mucosal lymphocyte cytokine profiles in children with autism and Gastrointestinal symptoms: mucosal immune activation and reduced counter regulatory Interleukin-10. J Clin Immunol. 2004;24(6):664–73. 15622451 10.1007/s10875-004-6241-6

[39] Ashwood P, et al. In search of cellular immunophenotypes in the blood of children with autism. PLoS ONE. 2011;6(5):e19299. 21573236 10.1371/journal.pone.0019299 PMC3087757

[40] DiStasio MM, et al. T lymphocytes and cytotoxic astrocyte blebs correlate across autism brains. Ann Neurol. 2019;86(6):885–98. 31591744 10.1002/ana.25610 PMC7210715

[41] Moaaz M, et al. Th17/Treg cells imbalance and their related cytokines (IL-17, IL-10 and TGF-β) in children with autism spectrum disorder. J Neuroimmunol. 2019;337:577071. 31671361 10.1016/j.jneuroim.2019.577071

[42] Bryn V, et al. Cytokine profile in autism spectrum disorders in children. J Mol Neurosci. 2017;61(1):1–7. 27730473 10.1007/s12031-016-0847-z

[43] Warren RP, et al. Immune abnormalities in patients with autism. J Autism Dev Disord. 1986;16(2):189–97. 2941410 10.1007/BF01531729

[44] Ahmad SF, et al. Dysregulation of Th1, Th2, Th17, and T regulatory cell-related transcription factor signaling in children with autism. Mol Neurobiol. 2017;54(6):4390–400. 27344332 10.1007/s12035-016-9977-0

[45] Ahmad SF, et al. Downregulation in helios transcription factor signaling is associated with immune dysfunction in blood leukocytes of autistic children. Prog Neuropsychopharmacol Biol Psychiatry. 2018;85:98–104. 29698674 10.1016/j.pnpbp.2018.04.011

[46] Sun L, Jin H, Li H. GARP: a surface molecule of regulatory T cells that is involved in the regulatory function and TGF-β releasing. Oncotarget. 2016;7(27):42826–36. 27095576 10.18632/oncotarget.8753 PMC5173174

[47] Edwards JP, et al. Regulation of the expression of GARP/Latent TGF-β1 complexes on mouse T cells and their role in regulatory T cell and Th17 differentiation. J Immunol. 2013;190(11):5506–15. 23645881 10.4049/jimmunol.1300199 PMC3668701

[48] Lehmkuhl P, et al. Dysregulated immunity in PID patients with low GARP expression on Tregs due to mutations in LRRC32. Cell Mol Immunol. 2021;18(7):1677–91. 34059789 10.1038/s41423-021-00701-z PMC8245512

[49] Zhang Y, et al. GP96 is a GARP chaperone and controls regulatory T cell functions. J Clin Invest. 2015;125(2):859–69. 25607841 10.1172/JCI79014 PMC4319419

[50] Tran DQ, et al. GARP (LRRC32) is essential for the surface expression of latent TGF-β on platelets and activated FOXP3+ regulatory T cells. Proc Natl Acad Sci. 2009;106(32):13445–50. 19651619 10.1073/pnas.0901944106 PMC2726354

[51] Tian J, et al. The role of GITR/GITRL interaction in autoimmune diseases. Front Immunol. 2020;11:588682. 33163004 10.3389/fimmu.2020.588682 PMC7581784

[52] Azuma M. Role of the Glucocorticoid-Induced TNFR-Related protein (GITR)-GITR ligand pathway in innate and adaptive immunity. Crit Rev Immunol. 2010;30(6):547–57. 21175417 10.1615/critrevimmunol.v30.i6.40

[53] Nocentini G, et al. *Expansion of regulatory GITR*^+^*CD25low/-CD4+ T cells in systemic lupus erythematosus patients*. Arthritis Res Therapy. 2014;16(5):444. 10.1186/s13075-014-0444-x PMC4209023 25256257

[54] Low frequency of GITR+ T cells in ex vivo and in vitro expanded Treg cells from type 1 diabetic patients | International Immunology | Oxford Academic. 10.1093/intimm/dxt020 23929911

[55] Shimizu J, et al. Stimulation of CD25^+^CD4+ regulatory T cells through GITR breaks immunological self-tolerance. Nat Immunol. 2002;3(2):135–42. 11812990 10.1038/ni759

[56] Ephrem A, et al. Modulation of Treg cells/T effector function by GITR signaling is context–dependent. Eur J Immunol. 2013;43(9):2421–9. 23722868 10.1002/eji.201343451 PMC11022273

[57] Douse CH, et al. TASOR is a pseudo-PARP that directs HUSH complex assembly and epigenetic transposon control. Nat Commun. 2020;11(1):4940. 33009411 10.1038/s41467-020-18761-6 PMC7532188

[58] Mathias B, et al. MYSM1 attenuates DNA damage signals triggered by physiologic and genotoxic DNA breaks. J Allergy Clin Immunol. 2024;153(4):1113–e11247. 38065233 10.1016/j.jaci.2023.12.001 PMC11417613

[59] Ostendorff HP, et al. Functional characterization of the gene encoding RLIM, the corepressor of LIM homeodomain factors. Genomics. 2000;69(1):120–30. 11013082 10.1006/geno.2000.6311

[60] Šikrová D, et al. SMCHD1 and LRIF1 converge at the FSHD-associated D4Z4 repeat and LRIF1 promoter yet display different modes of action. Commun Biology. 2023;6(1):677. 10.1038/s42003-023-05053-0 PMC10307901 37380887

[61] Ohkura N, Kitagawa Y, Sakaguchi S. Development and maintenance of regulatory T cells. Immunity. 2013;38(3):414–23. 23521883 10.1016/j.immuni.2013.03.002

[62] Deng G, et al. Foxp3 Post-translational modifications and Treg suppressive activity. Front Immunol. 2019;10:2486. 31681337 10.3389/fimmu.2019.02486 PMC6813729

[63] Lu J, et al. Metabolic controls on epigenetic reprogramming in regulatory T cells. Front Immunol. 2021;12:728783. 34421930 10.3389/fimmu.2021.728783 PMC8374078

[64] Frye RE. Editorial: early metabolic drivers of neurodevelopmental disorders: potential pathways to early detection and novel interventions. J Am Acad Child Adolesc Psychiatry. 2025. 10.1016/j.jaac.2025.11.015. 41338336 10.1016/j.jaac.2025.11.015

[65] Giulivi C, Napoli E, Schwartzer J, Careaga M, Ashwood P. Gestational exposure to a viral mimetic Poly(I:C) results in Long-Lasting changes in mitochondrial function by leucocytes in the adult offspring. Mediat Inflamm. 2013;2013:1–8. 10.1155/2013/609602. 10.1155/2013/609602 PMC3793312 24174710

[66] Hughes HK, Rowland ME, Onore CE, Rogers S, Ciernia AV, Ashwood P. Dysregulated gene expression associated with inflammatory and translation pathways in activated monocytes from children with autism spectrum disorder. 2022;12(1):39. 10.1038/s41398-021-01766-0 PMC8791942 35082275

[67] Li M, Kui X, Yang S, Nie Z, Chen H, Yao P, Xu X, Shen C, Li Z, Zhao H, et al. LdIL-2 treatment in ASD: a novel immunotherapeutic approach targeting Th/Treg dysfunction and neuroinflammation. Translational Psychiatry. 2025;15(1):376. 10.1038/s41398-025-03609-8. 41053009 10.1038/s41398-025-03609-8 PMC12500982

