# Supplementary material for: Altered phenotype and gene expression of regulatory T cells (Tregs) in children with Autism, and the relationship with comorbid gastrointestinal symptoms
Source: J Neuroinflammation. 2026 Feb 17;23:97. doi: 10.1186/s12974-026-03701-w (PMC13014816; doi:10.1186/s12974-026-03701-w)
Supplement: Supplementary file 3 — Supplementary Material 3. [file 12974_2026_3701_MOESM3_ESM.docx]

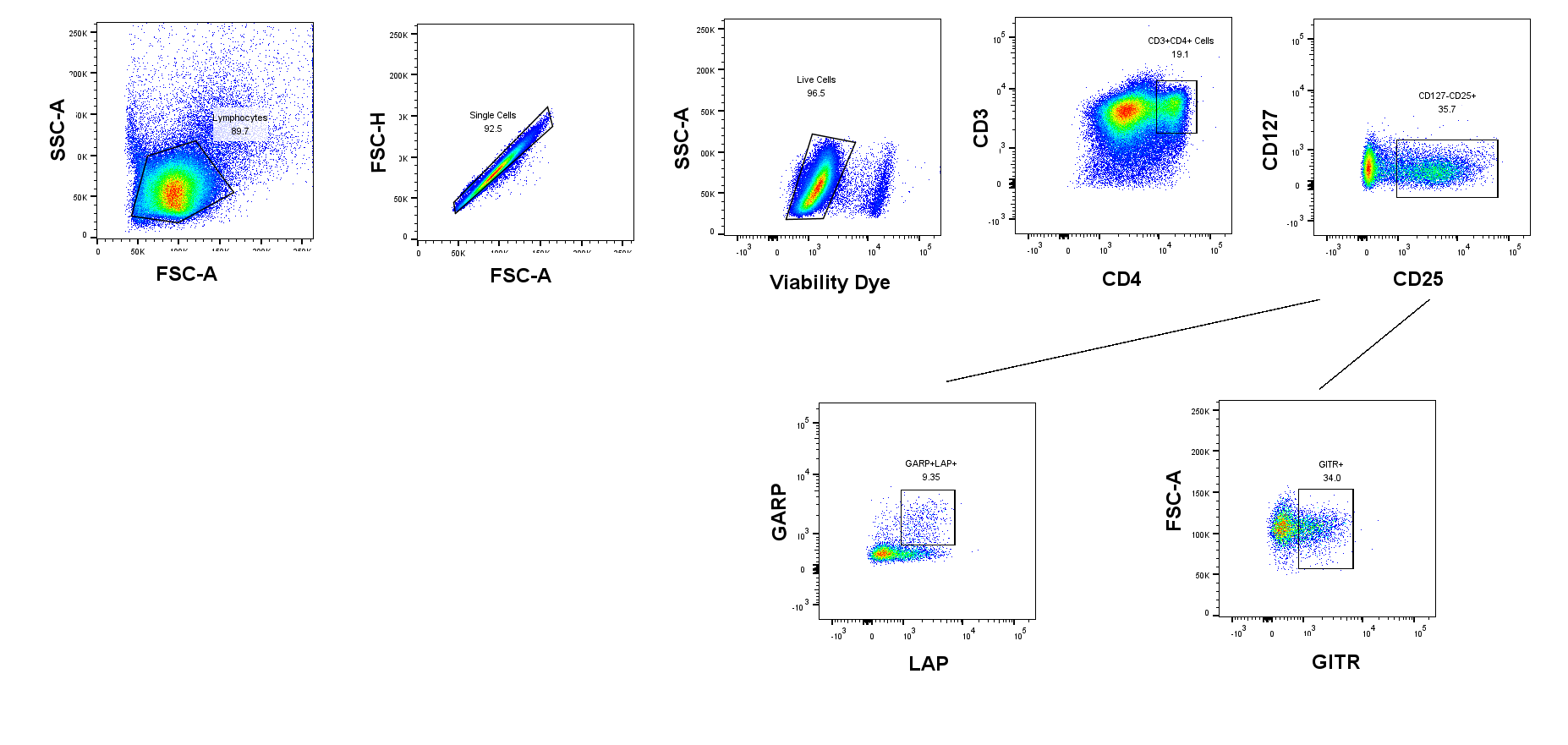


Supplemental Figure 1-Gerneral flow cytometry gating strategy used to identify Treg (CD3
+CD4+CD127-CD25+) from other T cell populations prior to downstream analysis
